# Beneficial effect of chronic *Staphylococcus aureus* infection in a model of multiple sclerosis is mediated through the secretion of extracellular adherence protein

**DOI:** 10.1186/s12974-015-0241-8

**Published:** 2015-02-03

**Authors:** Prateek Kumar, Benedikt Kretzschmar, Sabine Herold, Roland Nau, Mario Kreutzfeldt, Sandra Schütze, Mathias Bähr, Katharina Hein

**Affiliations:** Department of Neurology, University Medicine Goettingen, Robert-Koch-Strasse 40, 37075 Goettingen, Germany; Institute of Neuropathology, University Medicine Goettingen, Goettingen, 37075 Germany; Department of Pathology and Immunology, Centre Médical Universitaire 1, Rue Michel-Servet 1211, Geneva 4, Switzerland

**Keywords:** Infection, Experimental autoimmune encephalomyelitis, *S. aureus*, Eap, Neurodegeneration

## Abstract

**Background:**

Bacterial infections have been assumed to worsen multiple sclerosis (MS) disease symptoms and to lead to increased neurodegeneration. However, the underlying biological mechanisms for these effects are complex and poorly understood. Here, we assessed the disease-modulating effects of chronic infection with *Staphylococcus aureus*, a common human pathogen, on the clinical course and the extent of neurodegeneration in experimental autoimmune encephalomyelitis (EAE), an animal model of MS.

**Methods:**

To conduct this study, we established a persistent chronic infection in female brown Norway rats by inoculating *Staphylococcus aureus* (*S. aureus*) bacteria in a subcutaneously implanted tissue cages.

**Results:**

In this study, we observed that the introduction of a localized *S. aureus* infection during the subclinical phase of EAE induced a chronic systemic inflammatory response, consisting of increased T- and B-cell counts and systemic production of proinflammatory cytokines. Unexpectedly, the *S. aureus* infection completely prevented the development of clinical EAE, and markedly reduced inflammatory infiltration and demyelination of the optic nerve, while it increased the number of surviving retinal neurons. Using a *S. aureus* strain that lacked the extracellular adherence protein (Eap), we determined that the extracellular adherence protein is at least partially responsible for the inhibitory effect of *S. aureus* infection on autoimmune inflammation of the central nervous system.

**Conclusions:**

Our results demonstrate for the first time that chronic infection with *S. aureus* has a beneficial effect on EAE, indicating a dual role of infection in the pathogenesis of MS. We also showed that secretion of Eap by *S. aureus* plays a major role in preventing autoimmune inflammation of the CNS. Moreover, we identified Eap as a factor responsible for this protective effect.

## Background

Multiple sclerosis (MS) is an autoimmune inflammatory demyelinating disease of the central nervous system (CNS), which often manifests with optic neuritis in its early phase. Although the exact cause of this highly complex disease is still unknown, genetic and environmental factors are thought to be involved in disease initiation and pathogenesis [1]. A genome-wide study revealed an overrepresentation of immunologically relevant genes in MS patients [2]. However, the high rate of discordance in monozygotic twins [3] and data from migration studies indicate that environmental factors play an equal or even more important role than genes in the pathogenesis of MS.

Previous experimental and clinical studies demonstrated that bacterial and viral infections can trigger and aggravate autoimmune diseases [4-8]. Paradoxically, some microorganisms seem to prevent MS [9-11]. Thus, there is ongoing debate on the dual role of infection in the pathogenesis of MS. Moreover, the underlying biological processes, especially the association of systemic infection and neurodegeneration, a major histopathological correlate of disability in MS patients, are poorly understood. Given the complexity of the interplay between infection and autoimmunity, the majority of the knowledge stems from animal studies in experimental autoimmune encephalomyelitis (EAE), an animal model of MS.

In our present study, we induced EAE by immunization with myelin oligodendrocyte glycoprotein (MOG). Although the extent of spinal cord lesions in this model shows a certain variability, active immunization of female brown Norway (BN) rats with MOG leads to optic neuritis with acute axonal degeneration of the optic nerve (ON) and consecutive apoptosis of retinal ganglion cells (RGCs) in 90% of the animals [12-15]. Therefore, this EAE model provides a unique tool for investigating the morphological changes in neuronal cell bodies and in axons during neurodegeneration of the optic nerve.

*Staphylococcus aureus* (*S. aureus*) is a harmful pathogen in both hospital- and community-associated infections [16]. Locally applied *S. aureus* in implanted tissue cages have been found to induce persistent chronic systemic infections in animals [17]. In this study, we combined a newly established chronic *S. aureus* infection model with MOG-induced EAE in BN rats to investigate the impact of chronic systemic infection on the clinical course of MS and neurodegeneration in an animal model of MS.

## Methods

### Rats

Female brown Norway rats 8 to 10 weeks of age were used in all experiments. They were obtained from Charles River (Sulzfeld; Germany) and kept under environmentally controlled and pathogen-free conditions. All experiments involving animal use were performed in accordance with the relevant laws and institutional guidelines. These experiments have been approved by the local authorities of Braunschweig, Germany.

### Bacterial inoculum preparation

The *S. aureus* strains ATCC 29213 (kindly provided by Raimund Lugert from the Department of Microbiology, University of Goettingen; Germany), Newman ATCC 25904 [18] and the extracellular adherence protein (Eap) deficient strain of ATCC 25904, AH12 [19] (kindly provided by Markus Bischoff from the Institute for Medical Microbiology and Hygienie, University of Saarland; Germany) were used in these experiments. A single colony of the respective *S. aureus* strain was inoculated into brain heart infusion medium and incubated for 20 hours at 37°C in a shaking incubator. Afterwards, 50 μl of the bacterial culture grown for 20 hours were inoculated into 10 ml of fresh brain heart infusion medium and again incubated at 37°C in a shaking incubator. Bacteria were harvested after 6 hours of incubation at the time when the bacterial culture reached the log phase of growth. Bacterial concentrations were determined by quantitative plating on blood agar plates, and aliquots were kept at −80°C until further use.

### Retrograde labeling of retinal ganglion cells

Retrograde labeling of retinal ganglion cells (RGCs) and tissue cage implantation was done during a single anesthesia, 2 to 3 weeks prior to MOG immunization. Rats were anesthetized by intraperitoneal injection of ketamine (Ketanest 10; 0.95 ml/kg; Atarost, Twistringen; Germany) and xylazine 2% (0.25 ml/kg; Albrecht, Aulendorf; Germany) and positioned in a stereotaxic frame. The skin was incised mediosagittally, and holes were drilled into the skull above each superior colliculus (6.8 mm dorsal and 2 mm lateral from bregma). 2 μl of fluorescent dye fluorogold (5% in distilled water; Fluorochrome, Englewood, CO) were injected into both superior colliculi. After that, tissue cage implantation was performed.

### Experimental setup and timeline

**Figure 1:**
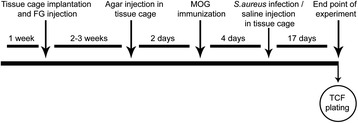
**Experimental timeline.** Tissue cage (TC) implantation and fluorogold (FG) injection was done 2 to 3 weeks prior to myelin oligodendrocyte glycoprotein (MOG) immunization. Agar was injected into the TC 2 days before immunization. Injection of *Staphylococcus aureus* (*S. aureus*) or saline was performed on day 4 postimmunization (pi). Animals were followed until day 8 of experimental autoimmune encephalomyelitis (EAE) or day 21 pi in case of clinically healthy animals. To monitor the infection, blood samples were taken throughout the whole experiment. Tissue was harvested from the optic nerve for histopathological analysis; retina was flat-mounted for quantification of retinal ganglion cells. Tissue cage fluid (TCF) was checked for the presence of bacteria.

Two experiments with a total of 62 animals were carried out (Figure 1). Tissue cage implantation was performed in all animals. Two to three weeks after the tissue cage implantation and retrograde labeling of RGCs, 400 μl of 2.3% semi-solid agar was injected into the tissue cage using a 27G needle (BD Eclipse, Heidelberg; Germany) in order to enable persistent infection throughout the course of the experiment by providing extra surface area for the growth of bacteria. Before the agar injection, tissue cage fluid (TCF) was obtained percutaneously from all animals and checked for sterility by plating on blood agar plates. Animals were excluded from the experiment if they had contaminated TCF. Two days after the agar injection into the tissue cage, MOG-immunization was done and considered day zero of immunization.

A rapid neurodegeneration occurs already in the preclinical phase of EAE [14], which causes the failure of most of therapeutic interventions, when applied at later time points during the disease course [15,20]. Therefore, infection was performed on day 4 postimmunization (pi) to elucidate whether infection modulates the course of EAE and has any influence on the neurodegeneration in this animal model. In the first experiment (n = 36), one animal group was infected with a wild-type strain of *S. aureus* (ATCC 29213). The corresponding control group received saline. To prove our hypothesis of the anti-inflammatory effect of Eap, animals (n = 26) were randomized into the three different groups. One group was infected with the wild-type *S. aureus* strain (ATCC 25904). The second group was infected with the *S. aureus* strain deficient for Eap (AH12). The corresponding control group received saline. Animals were followed until day 8 after clinical manifestation of the disease (EAEd8) or day 21 pi in case of clinically healthy animals. At the end of the experiment, TCF was obtained percutaneously from all (infected and noninfected) animals. TCF was checked for the presence of bacterial colonies by plating on blood agar plates.

### Tissue cage preparation and implantation

Rigid polytetrafluoroethylene (teflon) tissue cages (internal and external diameter of 8 and 10 mm respectively, length 25 mm) perforated by around 100 regularly spaced holes (diameter 1 mm) containing two polymethylmethacrylate (PMMA) cover slips per tissue cage, sealed at both ends with a suture (Ethicon, Norderstedt; Germany), were used as a foreign body implant. A cutaneous incision in the left flank was made under anesthesia, and one autoclaved tissue cage was subcutaneously implanted under sterile conditions into each animal.

### Induction of chronic infection

Infection was performed by injection of 200 μl of bacterial culture diluted in 0.9% NaCl (saline) (B. Braun, Melsungen; Germany) containing 2 × 10^7^ colony forming units (CFU) of the respective *S. aureus* strain into the agar of the tissue cage. Control animals received 200 μl saline into their tissue cage.

### Induction and evaluation of experimental autoimmune encephalomyelitis

Rats were anesthetized by inhalation anesthesia with isoflurane (Abbott, Wiesbaden; Germany) and injected intradermally at the base of the tail with a total volume of 200 μl inoculum, containing 50 μg recombinant rat MOG^Igd^ (kindly provided by C. Stadelmann, Institute of Neuropathology, Goettingen; Germany) in saline emulsified (1:1) with complete Freund’s adjuvant (CFA; Sigma-Aldrich, St. Louis, MO) containing 200 μg heat-inactivated *Mycobacterium tuberculosis* (strain H 37 RA; Difco Laboratories, Detroit, MI). Rats were scored for clinical signs of EAE and were weighed daily. This score reflects the amount of spinal cord lesions and does not include visual symptoms or correlate with the severity of optic neuritis ([15,21]. The signs were scored as follows: grade 0, no symptoms; grade 0.5, distal paresis of the tail; grade 1, complete tail paralysis; grade 1.5, paresis of the tail and mild hind limb paresis; grade 2.0, unilateral severe hind limb paresis; grade 2.5, bilateral severe hind limb paresis; grade 3.0, complete bilateral hind limb paralysis; grade 3.5, complete bilateral hind limb paralysis and paresis of one front limb; grade 4, complete paralysis (tetraplegia), moribund state, or death.

### Leukocyte count

To monitor the systemic infection leukocytes were counted in the peripheral blood every second day until day 8 after MOG immunization and then at intervals of 3 days until day 20 pi. Blood samples were taken sublingually after inhalation anesthesia with isoflurane. A total of 10 μl of blood sample was diluted in 190 μl of 4% glacial acetic acid, and leukocyte counting was performed in a Neubauer-haemocytometer (Superior-Marienfeld, Lauda-Königshofen; Germany).

### Western blotting

For Western blot analysis, 6 animals (n = 3 for each group) were sacrificed on day 3 postinfection. Blood samples were collected by cardiac puncture using 18 G needle (BD Eclipse, Heidelberg; Germany) from *S. aureus* infected and noninfected animals and placed on ice for 30 minutes. Serum was separated from the whole blood by centrifuging it at 3,500 rpm for 10 minutes, and protein concentration of the serum was measured using BCA assay kits (Thermo Fisher Scientific Inc; U.S.A). Equal amount of protein from each sample was subjected to 12% SDS-PAGE and transferred onto a nitrocellulose membrane. The membrane was blocked with 5% nonfat dry milk in Tris-buffered saline containing 0.1% Tween 20 (TBST) for 1 hour at room temperature. Incubations with rabbit anti-Eap antibody (1:1500; Cat# ab92982, Abcam; U.K.), was performed overnight at 4°C. After being washed, the membrane was incubated with a horseradish peroxidase-conjugated appropriate secondary antibody (1:5,000 Jackson Immuno Research Europe; U.K.) in 5% nonfat dry milk in TBST for 1 hour at room temperature, and immune-reactive proteins were detected using the enhanced chemiluminescence method (ECL; Amersham). Also, 3 μg of purified Eap (a gift from Markus Bischoff, University of Saarland, Germany) was used as positive control. Ponceau staining was used to confirm equal loading of proteins.

### Histopathology and immunohistochemistry

Retinas were removed for counting of RGCs, and paraformaldehyde-fixed paraffin-embedded optic nerves (ONs) were taken for histopathological evaluation. After perfusion of rats with 4% PFA, ONs were post-fixed in 4% PFA for 24 h and embedded in paraffin. Histological evaluation was performed on 2 μm thick slices stained with Luxol Fast Blue to assess demyelination. Photos of vertical sections were taken using an AxioCam MR Microscopy camera (Zeiss, Göttingen; Germany). The images were processed using Zeiss AxioVision 4.2 software to evaluate the demyelinated area as a percentage of the whole ON cross section. Immunohistochemistry was performed on paraffin embedded ON cross sections representing three different levels of an ON. ED1 macrophages/ activated microglia (AbD Serotec Cat# MCA341R RRID: AB_2291300), were detected in cross sections using avidin-biotin detection. Spleen sections served as positive control for ED1 staining. ED1 positive cells were evaluated using the following score: 0, no labeled cells; 1, a few ED1 positive cells (number countable, infiltration of less than 10% of the ON cross-section area); 2, infiltration of less than 10% of the ON cross section area with ED1 positive cells in at least one of the three cross sections of an ON, number of ED1 positive cells not countable; 3, infiltration of 10 to 50% of the ON cross section area with labeled ED1 positive cells in at least one of three cross sections of an ON; 4, infiltration of 50 to 80% area of the ON cross-section area in at least one of the three cross sections of an ON; and 5, infiltration of more than 80% of the ON cross section area in at least one of the three cross sections of an ON.

### Quantification of T cell infiltrates in optic nerve

CD3 staining for assessment of infiltration of CD3-positive cells across the blood–brain barrier was performed on 0.5 μm thick cross section slices of paraffin-embedded ONs using anti-CD3 antibody (Biozol Cat# BZL03543 RRID: AB_2313498). CD3-positive cells were detected by biotin-avidin detection. Spleen sections served as positive control for CD3 staining. To quantify T-cell infiltrates in the ON, we used tissue studio that is based on *Cognition Network Language* (Definiens Cognition Network Technology®, Definiens Developer XD software, Munich; Germany) In brief, CD3-stained sections were scanned using the 3D Histech 250 Flash slide scanner at 200-fold magnification. The area of interest (optic nerve) was first detected from the background by the color spectrum. After segmentation, nuclei and CD3-positive cytoplasm were discriminated. Within each area of interest, the total number of CD3-positive cells was calculated and expressed as cells/mm^2^.

### Quantification of axonal densities in optic nerve

To quantify axonal densities in the ON, 0.5-μm thick cross section slices of ONs were stained by Bielschowsky silver impregnation staining to visualize axonal structures. Automated quantification of axonal densities was performed on high magnification pictures (100x) applying a custom-programmed script in *Cognition Network Language* based on the Definiens Cognition Network Technology® platform (Definiens Developer XD software, Munich; Germany). Briefly, the programmed script first discriminates between tissue and background (no tissue) by spectral difference detection. Subsequently, the area of detected tissue (region of interest, ROI) was calculated and ‘axonal structures’ within this ROI were detected based on their dark black color. To discriminate between nucleoli of inflammatory cells and axons (which both structures are stained black in Bielschowsky silver impregnation) only black structures without a faint brown-stained circumference (nucleus) were further classified as axonal structures. In a next step, multiple axons in direct contact with each other were further split into the corresponding individual axons if the splitting improved the elliptic shape of an axon. Within each area ROI, the total number of axons was calculated. The density of axons in each ON was measured in at least nine standardized microscopic fields of each ON. The mean axon density was calculated for each ON and expressed as axons/mm^2^.

### Quantification of retinal ganglion cell density

Retinae were dissected, flat-mounted on glass slides, and examined by fluorescence microscopy (Zeiss Axioplan 2) using a DAPI filter (315/395 nm). Retinal ganglion cell densities were determined by counting labeled cells in three areas (62,500 μm^2^ each) per retinal quadrant at three different eccentricities of 1/6, 3/6, and 5/6 of the retinal radius. Cell counts were performed by two independent investigators following a blinded protocol.

### Flow cytometry

Flow cytometry for lymphocyte counting was performed in blood on day 4 pi prior to infection and 2 days after infection at the time when the leukocyte counts reached their peak. In brief, 100 μl blood taken sublingually was collected in 250 μl of 5 mM EDTA prepared in isotonic PBS. Samples were washed once with PBS, and RBC lysis buffer (0.1 M ammonium chloride in PBS) was added in each sample. Samples were left at 4°C for 10 minutes and washed once with PBS. A total of 100 μl of FACS buffer (PBS + 1% BSA + 0.1% NaN_3_) containing anti-CD45RA (BioLegend Cat# 202307 RRID: AB_314010) and anti-CD3 (BioLegend Cat# 201408 RRID: AB_893304) antibodies were added in each samples at a dilution of 1:200. Incubation was done for 45 minutes at 4°C in the dark. The cells were washed once with PBS and re-suspended in 200 μl of FACS buffer prior to flow cytometric analysis. Analysis was performed by four-color flow cytometry using a FACSCalibur and FacScan software (BD; Goettingen; Germany).

### Luminex assay

A Luminex assay kit (Bio-Plex Pro™ Rat Cytokine Th1/Th2 Assay #171-K1002M), containing premixed coupled magnetic beads and detection antibodies for detecting 12 rat cytokines (IL-1 alpha, IL-1 beta, IL-2, IL-4, IL-5, IL-6, IL-10, IL-12, IL-13, TNF-alpha, INF-gamma, GM-CSF) was used for measurement of cytokine concentrations in serum samples collected at the end of the experiment. The total number of animals used in this cytokine assay was 25 (n = 11 infected with wild-type strain of *S. aureus* (ATCC 25904); n = 9 infected with Eap-deficient of *S. aureus* (AH12); n = 5 noninfected control). Quantification of cytokines was performed according to the manufacturer’s instructions. Measurement was performed by using a Bioplex 200-system based on the Luminex xMAP-technology (Bio-Rad, Hercules, CA, USA). The signal detection occurred using the Bioplex 200-system and the Bioplex Manager Software version 6.0.

### Statistical analysis

Data are presented as means ± SEM. Student’s *t*-test was used to analyze differences between two animal groups. Multiple comparisons were statistically evaluated by one-way ANOVA using Prism 5 (GraphPad Software). Mann–Whitney U test was used for the analysis of the EAE score (SPSS, IBM). A *P* value of <0.05 was considered to be statistically significant.

## Results

### Local infection with *Staphylococcus aureus* leads to persistent systemic inflammation

**Figure 2:**
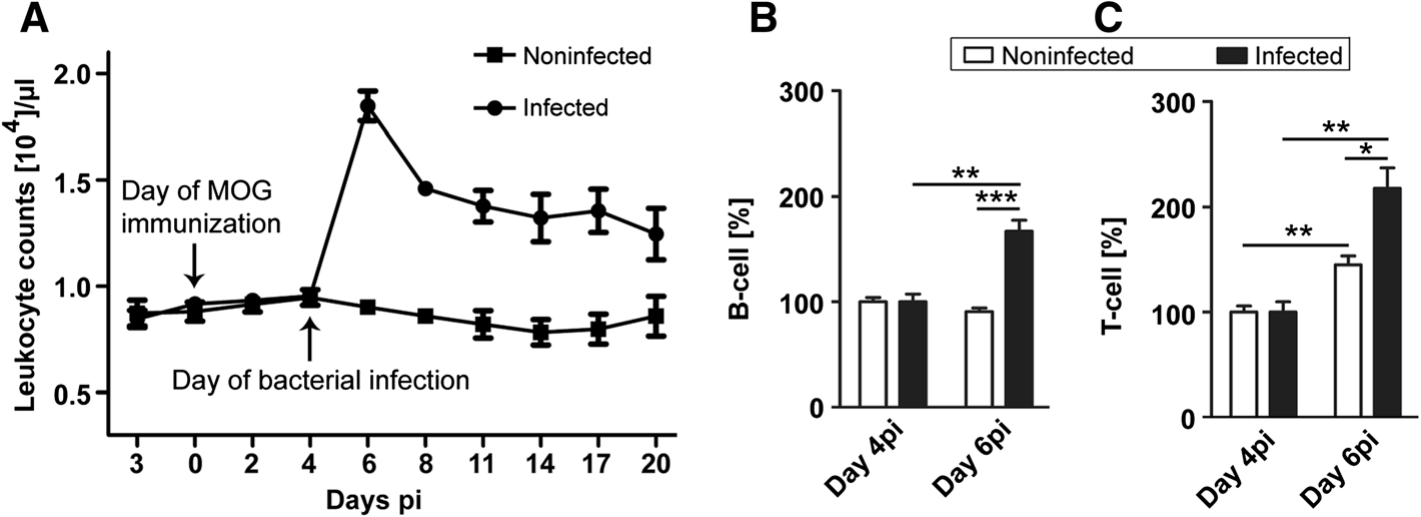
**Leukocyte counting and flow cytometry.** Leukocyte counts **(A)** were performed in peripheral blood of infected and noninfected myelin oligodendrocyte glycoprotein (MOG)-immunized animals. The leukocyte count was found to be significantly higher in the infected group. Data are represented as means ± SEM of all animals from each group (n = 12, infected; n = 11, noninfected). Flow cytometry was performed in peripheral blood to assess B- and T-cell populations in animals infected with *S. aureus* and noninfected control group on day 4 postimmunization (pi) (prior to infection) and on day 6 pi (corresponding to day 2 postinfection). B- **(B)** and T-cell **(C)** populations were substantially increased in infected animals 2 days after infection. Data are presented as means ± SEM. (n = 4 in each group; *P <0.05; **P <0.01; ***P <0.001).

To confirm the persistent local infection in the animals, we collected tissue cage fluid (TCF) from all animals under sterile conditions at the end of the experiment and plated the TCF on blood agar plates. TCF obtained from infected animals contained a high number of bacteria (*S. aureus*), whereas no bacterial growth was observed in TCF from noninfected control animals. Due to the semisolid consistency of TCF, which is probably due to the presence of agar, dilutions for a more exact determination of bacterial numbers were not possible. To ensure that local application of bacteria induced systemic inflammation, leukocytes were counted in peripheral blood samples until day 20 pi (corresponding to day 16 postinfection). Here, we observed that the leukocyte counts in infected animals (18475 ± 685/μl; n = 12; Figure 2A) were significantly elevated 2 days after infection compared to noninfected control animals (9000 ± 305/μl; n = 11; *P* <0.001; Figure 2A). Afterwards, leukocyte counts decreased in the infection group, but remained significantly elevated compared to the control group until day 20 pi (12457 ± 1219/μl versus 8583 ± 936/μl, *P =* 0.03; Figure 2A).

An anti-inflammatory effect of Eap secreted by *S. aureus* was demonstrated in chronic infections as well as in autoimmune disease models [22-24]. Therefore, we hypothesized that this protein is responsible for the protective effects of *S. aureus* infection in EAE. To prove our hypothesis, a second set of experiments was performed by including a group of animals infected with a *S. aureus* strain deficient for Eap. In this experiment, leukocyte counts were significantly elevated two days after infection in the animals infected with the wild-type strain (17236 ± 742/μl; n = 11; *P <*0.001) and in the animals infected with the Eap-deficient strain (18575 ± 585/μl; n = 10; *P <*0.001) in contrast to the control group (9940 ± 527/μl; n = 5) (data not shown). We did not observe any significant difference between the leukocyte counts of the two infection groups at any time point during the whole course of the experiment.

### Infection with *Staphylococcus aureus* enhances T- and B-cell counts in peripheral blood

Flow cytometry was performed to assess the B- and T-cell counts in peripheral blood on day 4 post immunization (pi) (directly prior to infection) and day 6 pi (corresponding to day 2 after the infection). B-cell counts (Figure 2B; n = 4 in each group) were significantly increased in the *S. aureus*-infected group (67 ± 10.55%; *P* = 0.002) on day 6 pi compared to day 4 pi. In contrast, no significant change in B-cell counts was observed in noninfected animals on day 6 pi compared to day 4 pi. T-cell counts (Figure 2C; n = 4 in each group) increased significantly in both groups on day 6 pi compared to day 4 pi, however, the increase was more prominent in infected than in noninfected animals (117 ± 19.38% versus 45 ± 8.32%; *P* = 0.013; Figure 2C). These results show that infection with *S. aureus* induces both B-cell and T-cell response in peripheral blood.

### Infection with *Staphylococcus aureus* leads to increased concentrations of pro-inflammatory cytokines in the systemic circulation

**Figure 3:**
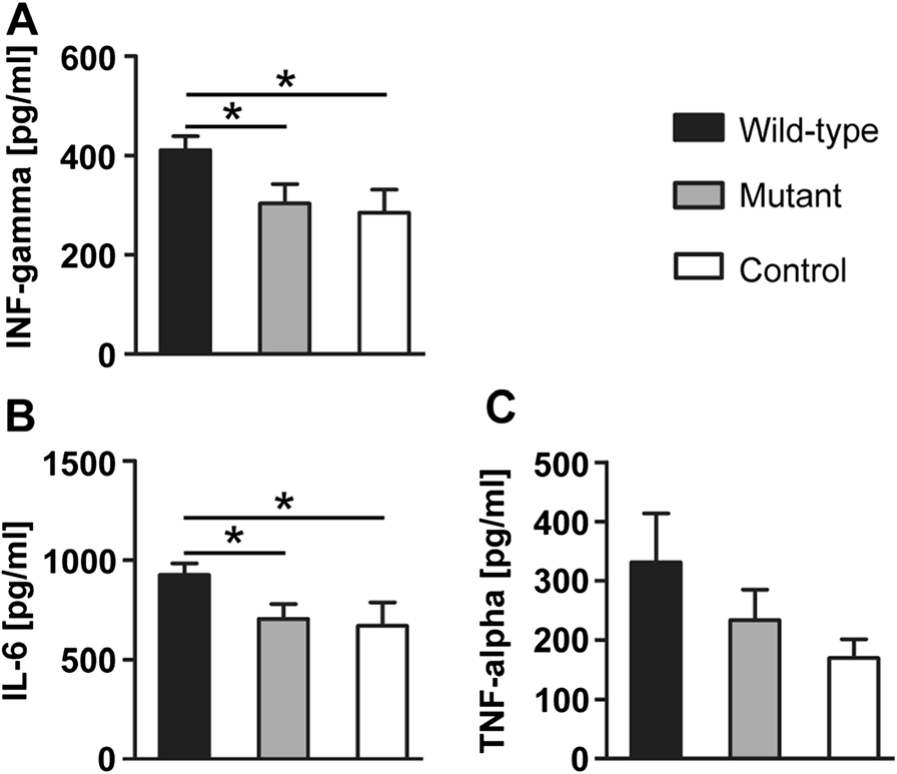
.**Luminex assays.** Luminex detection assays indicated a significant increase of IFN-gamma **(A)** and IL-6 **(B)** in blood of animals infected with *Staphylococcus aureus* wild-type strain. TNF-alpha showed a tendency towards an increase of level in the group infected with the wild-type *S. aureus* **(C)** (n = 11, wild-type; n = 9, Eap-deficient (mutant); n = 5, saline treated (control); *P <0.05).

Among the 12 cytokines measured by Luminex assay in serum samples collected at the end of the experiment, we observed differences in the levels of IFN-gamma, IL-6 and TNF-alpha. More detailed, levels of IFN-gamma (Figure 3A) was significantly increased in animals infected with the wild-type bacteria (411.4 ± 28.37 pg/ml) compared to animals infected with the Eap-deficient strain (303.9 ± 39.08 pg/ml; *P* = 0.035) and compared to noninfected control animals (284.9 ± 46.49 pg/ml; *P* = 0.029). Serum levels of IL-6 (Figure 3B) were also significantly elevated in the group infected with the wild-type *S. aureus* bacterium (926.6 ± 57.27 pg/ml) compared to the group infected with the Eap-deficient bacterium (704.4 ± 75.48 pg/ml; *P* = 0.028) and the control group (668.7 ± 119.1 pg/ml; *P* = 0.042). A tendency towards higher serum levels of TNF-alpha (Figure 3C) was observed in the animals infected with wild-type *S. aureus* bacteria (330.7 ± 82.96 pg/ml) compared to the group infected with Eap-deficient *S. aureus* (233.2 ± 51.44 pg/ml; *P =* 0.35) and the noninfected control group (169.9 ± 30.89 pg/ml; *P =* 0.22). The level of other cytokines (IL-1 alpha, IL-1 beta, IL-2, IL-4, IL-5, IL-10, IL-12, IL-13 and GM-CSF) did not change significantly.

### *Staphylococcus aureus* infection prevents the manifestation of clinical symptoms and suppresses disease activity in myelin oligodendrocyte glycoprotein induced experimental autoimmune encephalomyelitis

**Figure 4:**
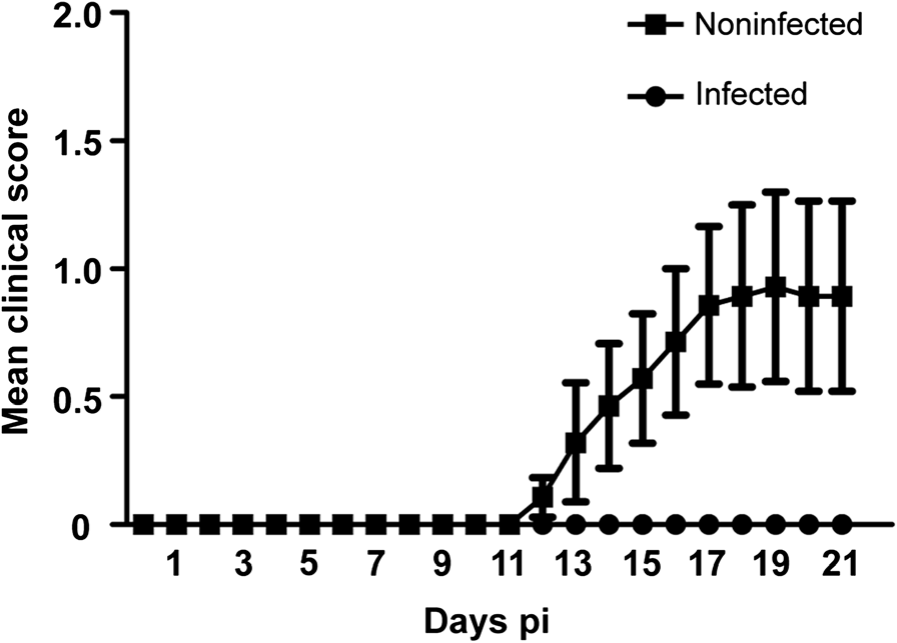
**Clinical score.** Infection with wild-type *Staphylococcus aureus* (ATCC 29213) on day 4 postimmunization (pi) prevented disease onset in myelin oligodendrocyte glycoprotein induced experimental autoimmune encephalomyelitis (MOG-EAE). Data represented as means ± S.E.M. of the daily score. The clinical score represents spinal cord lesions. Presence of optic neuritis is not included (n = 16, infected; n = 14, noninfected).

Animals were infected with *S. aureus* on day 4 after immunization to avoid interference with the initiation of the autoimmune response. Around day 13 after immunization, 6 of the 14 placebo-infected animals developed clinical symptoms while infection with wild-type *S. aureus* (ATCC 29213) prevented the clinical manifestation of EAE in all the infected animals. The mean cumulative score differed significantly between the group infected with *S. aureus* and the respective control group (*P* = 0.047; Figure 4). Concerning the clinical manifestation of the disease, a similar result was observed in the experiment in which the Eap-deficient strain and the corresponding wild-type strain were used. Although no clinical signs of EAE were observed in the group infected with *S. aureus* (ATCC 25904) or in the group infected with the Eap-deficient strain (AH12) of *S. aureus*, animals of the group infected with Eap-deficient *S. aureus* displayed level of histopathological abnormalities (see below). However, noninfected control animals were severely affected with a mean EAE score of 2.4 ± 0.36 (n = 5; *P* <0.001; data not shown here) at the end of the experiment.

### Infection with *Staphylococcus aureus* reduces neuropathological damage of the optic nerve and increases retinal ganglion cells survival after acute optic neuritis

At day 8 of clinical disease or at day 21 after immunization in the absence of manifest disease, we determined the extent of demyelination of the optic nerves (ONs) by Luxol Fast Blue staining. Additionally, we performed immunohistochemistry for ED1 macrophages/microglia and CD3^+^ T-lymphocytes to assess the extent of inflammatory infiltration. Chronic axonal damage was assessed by Bielschowsky’s silver impregnation. For each staining, ON cross sections of three different levels were evaluated.

**Figure 5:**
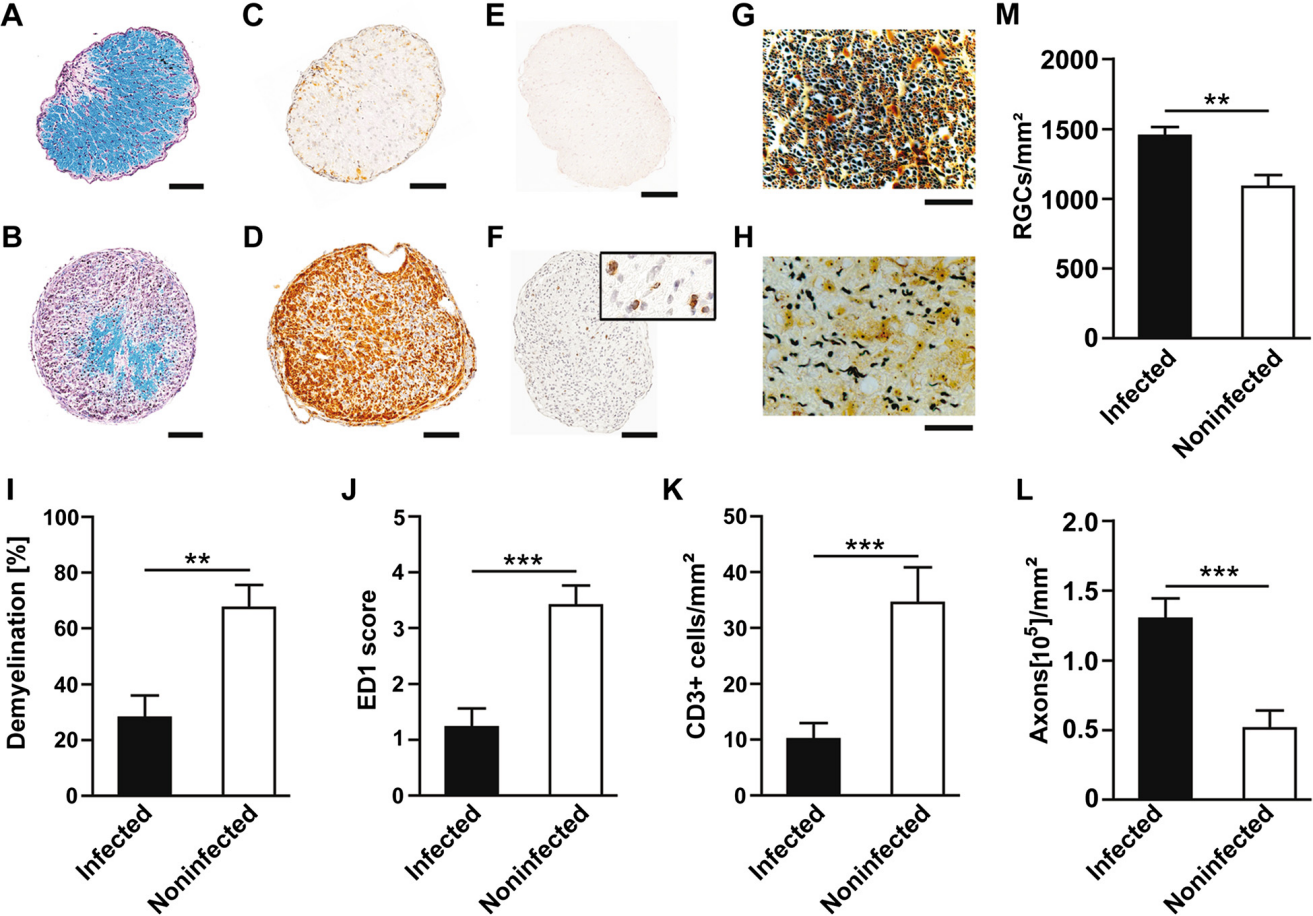
**Optic nerve (ON) histopathology and retinal ganglion cell (RGC) counts. A** and **B**: Representative Luxol Fast Blue-stained cross sections of ONs of a wild-type *Staphylococcus aureus* infected rat **(A)** showed only small areas of demyelination, with mainly intact myelin (blue); in contrast, a cross section from a noninfected control animal **(B)** showed extensively demyelinated areas (purple). **C** and **D**: Representative examples of the substantially higher number of ED1 macrophages/activated microglia detected in the ON of a noninfected control animal **(D)**, compared with an animal infected with wild-type *S. aureus* **(C)**. **E** and **F**: representative images for CD3^+^ T-lymphocyte counts, which were noted higher in the optic nerve of the animals from control group **(F)**, whereas CD3-positive cells were hardly present in the optic nerve of the group infected with bacteria **(E)**. **G** and **H**: Bielschowsky’s silver impregnation of ON cross sections revealed a higher density of axons on experimental autoimmune encephalomyelitis (EAE) day 8 in wild-type *S. aureus* infected animals **(G)** compared to noninfected control animals **(H)**. **I**, **J**, **K** and **L** represent the quantitative data for demyelination, ED1 score, CD3-positive cells and for axonal loss, respectively. **M**: Represents the quantitative data for fluorogold labeled RGCs, which showed higher density in the animals infected with *S. aureus* compared to the noninfected control group. For optic nerve histopathology, n = 16 infected, n = 14 noninfected; for RGC counts n = 20 infected; n = 14 noninfected, where n indicates the number of eyes used for counting the RGCs; **P <0.01; ***P <0.001). Bar length for Luxol Fast Blue, ED1, CD3- = 100 μm, and for Bielschowsky’s silver impregnation = 20 μm.

In the animals infected with the *S. aureus* wild-type strain, we observed significantly less demyelination in ON cross sections (28.08 ± 7.94%; Figure 5A, I; n = 16) compared to noninfected control animals (67.41 ± 8.17%; *P =* 0.0011; Figure 5B, I; n = 14). Analysis of inflammatory infiltrates with ED1 staining revealed a substantially lower infiltration by macrophages and activated microglia in the infected group (1.22 ± 0.33; Figure 5C, J; n = 16) compared to the control group (3.41 ± 0.35; *P* <0.001; Figure 5D, J; n = 14). Quantification of CD3^+^ cells revealed that only the minority of infiltrating cells were CD3^+^ T-lymphocytes. At day 21 after immunization, numbers of CD3^+^ cells were found to be significantly lower in the *S. aureus* infected group (10.09 ± 2.88/mm^2^; Figure 5E, K; n = 16) in comparison to the noninfected control group (34.46 ± 6.39/mm^2^; *P* <0.001; Figure 5F, K; n = 14). Using Bielschowky’s silver impregnation, we observed a higher axonal density in infected animals (129700 ± 14580/mm^2^; Figure 5G, L; n = 16) compared to noninfected control animals (51260 ± 12810/mm^2^; *P* <0.001; Figure 5H, L; n = 14).

To evaluate the impact of infection on neurodegeneration, numbers of surviving RGCs, the neurons that form the axons of the ON, were counted on day 8 of MOG-EAE. Previously, we have shown that in healthy BN rats sham-immunized with complete Freund’s adjuvant (CFA), 2730 ± 145 RGCs/mm^2^ were detectable (mean ± SEM; n = 8) [13]. In our present study, the mean RGC density in noninfected control animals at day 8 of MOG-EAE dropped to 1085 ± 85.7/mm^2^ (Figure 5M; n = 14). In contrast, RGC density in infected animals was significantly higher (1449 ± 64.60/mm^2^, *P* = 0.002; Figure 5M; n = 20). For RGCs counts, n indicates the number of eyes.

### Extracellular adherence protein is present in serum of animals infected with *Staphylococcus aureus*

**Figure 6:**
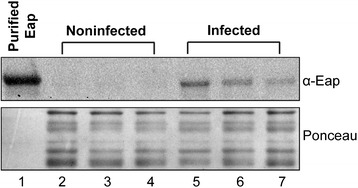
**Western blot analysis.** Western blot analysis showed the presence of extracellular adherence protein (Eap) in the serum of animals infected with wild-type *S. aureus* bacterium (Lane 5, 6 and 7). No Eap was detected in the serum samples of the control animals (Lane 2, 3 and 4). Purified Eap (Lane 1) was used as a positive control. Ponceau staining was used as a loading control.

To investigate the presence of Eap in the peripheral blood circulation of infected animals, Western blot analysis was performed in serum samples collected from rats infected with wild-type *S. aureus* as well as from MOG-immunized noninfected control animals. Western blot analysis revealed the presence of Eap in the circulation of the animals infected with *S. aureus* (Figure 6). No Eap was detected in the serum samples collected from the MOG-immunized noninfected control animals (Figure 6).

### The protective effect of wild-type S*taphylococcus aureus* infection on optic neuritis is reduced in the group infected with respective extracellular adherence protein-deficient strain

**Figure 7:**
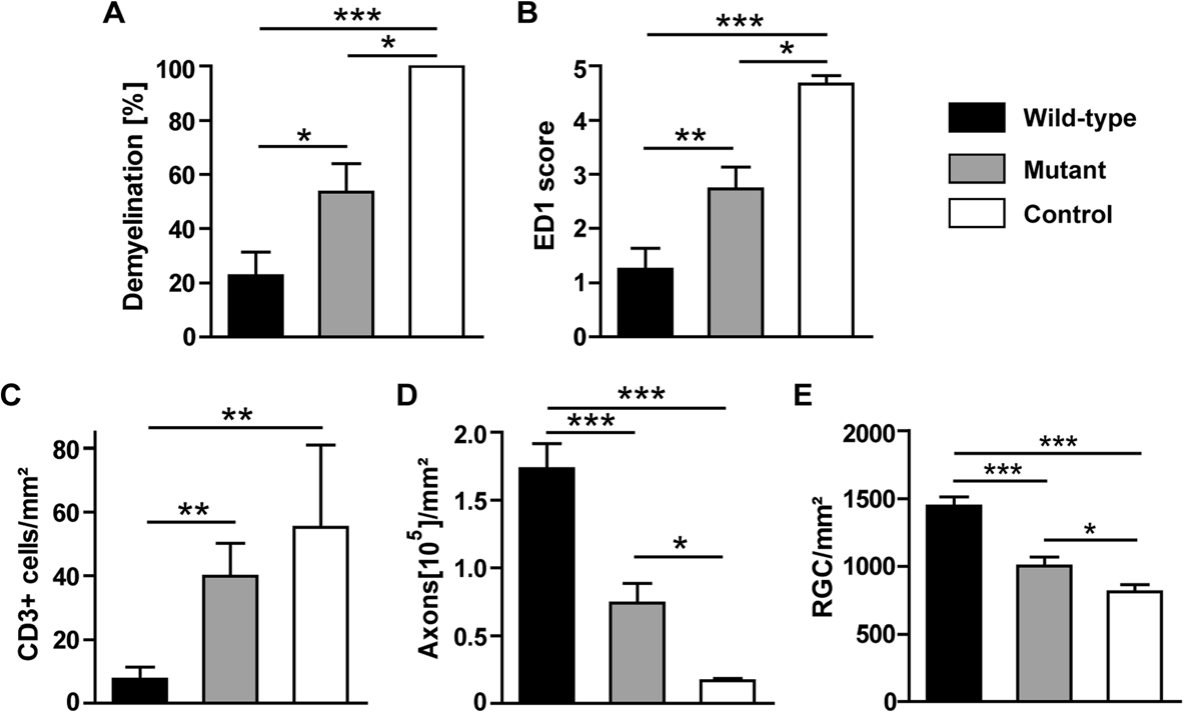
**Optic nerve (ON) histopathology and retinal ganglion cell (RGC) counts.** The positive effect of *S. aureus* on optic neuritis was reverted in the Eap-deficient strain. Quantitative data for extent of demyelination **(A)**, inflammation **(B, C)**, axonal damage **(D)** and RGC counts **(E)**. Animals infected with the wild-type strain of *S. aureus* (wild-type; n = 11), with the Eap deficient strain (mutant; n = 10) or treated with saline (control; n = 4); while for RGC counts (n = 18, wild-type; n = 13, mutant; n = 9, control), n indicates the number of eyes used for counting the RGCs), (*P <0.05; **P <0.01; ***P <0.001).

In further experiments, an Eap-deficient *S. aureus* strain and the respective wild-type *S. aureus* strain were used for infection of the animals. In these experiments, we observed significantly less demyelination (Figure 7A) in wild-type *S. aureus* infected animals (22.77 ± 8.46%; n = 11) in comparison to both Eap-deficient infected (53.53 ± 10.56%; *P* = 0.027; n = 10) and control animals (100 ± 0%; *P* <0.001; n = 4). Accordingly, the extent of inflammation accessed by ED1 staining (Figure 7B) was significantly lower in the group infected with wild-type *S. aureus* bacteria (1.24 ± 0.39; n = 11) in comparison to the group infected with Eap-deficient bacteria (2.73 ± 0.40; *P* = 0.012; n = 10) and the noninfected control group (4.66 ± 0.15; *P* <0.001; n = 4). CD3^+^ T-Lymphocyte counts revealed significantly lower numbers of CD3^+^ cells (Figure 7C) in animals infected with wild-type *S. aureus* (7.6 ± 3.75/mm^2^; n = 11) in comparison to the animals infected with Eap-deficient bacteria (39.81 ± 10.37/mm^2^; *P* = 0.004; n = 10) and the control group (55.2 ± 25.8/mm^2^; *P* = 0.007; n = 4). No significant difference was observed in CD3^+^ cell counts between the animals infected with Eap-deficient *S. aureus* bacteria and the noninfected control group (*P* = 0.5). Bielschowsky’s silver impregnation was performed to assess the axonal pathology of the optic nerve (Figure 7D) and revealed that the animals infected with wild-type bacteria showed a higher number of axonal counts (173500 ± 18220/mm^2^; n = 11) in comparison to the group infected with Eap-deficient bacteria (73953 ± 14500/mm^2^; *P* <0.001; n = 10) and noninfected control animals (16670 ± 1938/mm^2^; *P* <0.001; n = 4). The RGC density was higher in the animal group infected with the wild-type strain (1447 ± 66/mm^2^; Figure 7E; n = 18) compared to animals infected with the Eap-deficient strain (1001 ± 65/mm^2^; *P* <0.001; Figure 7E; n = 13) and the noninfected control group (810 ± 52/mm^2^, *P* <0.001; Figure 7E; n = 9).

## Discussion

Using a rat model of MOG-induced EAE, we have demonstrated for the first time that chronic infection with *S. aureus* prevents the onset of EAE and reduces the development of histopathologic changes in the optic nerve. Using an extracellular adherence protein (Eap)-deficient strain of *S. aureus*, we showed that secretion of Eap by *S. aureus* plays a major role in preventing inflammation of the central nervous system (CNS).

Peripheral infection has been shown to exacerbate the immune response in experimental models of MS and in patients [7,25-28]. However, investigations of the role of infection in MS patients are complicated by the lack of a surrogate marker of disease activity. The results of magnetic resonance imaging (MRI) studies on the influence of peripheral infection on the number of contrast-enhancing MS lesions as a marker of disease activity are inconsistent [26,29]. Moreover, reliable markers to assess neurodegeneration in MS patients do not exist. For these reasons, much of the knowledge regarding the relationship between systemic infection and autoimmunity in MS is derived from EAE model studies.

Previous EAE model studies have focused on analyzing the clinical course of the disease. These studies used bacterial compounds (for example, LPS, enterotoxins) [30-32] rather than whole viable bacteria; thus, their results do not accurately reflect the clinical situation of MS patients. To our knowledge, this study is the first to investigate the effects of chronic infection with viable bacteria in an EAE model. By locally injecting a *S. aureus* strain, which is pathogenic for humans, into a previously implanted tissue cage, we established a stable chronic systemic infection in BN rats. This infection model is advantageous because it induces a mild infection that does not require antibiotic treatment to keep the animals alive. In our study, two days after the local application of the bacteria, corresponding to day 6 after immunization, the systemic infection became prominent as determined by increased leukocyte counts in peripheral blood. The rationale for the selected infection time point came from experimental and clinical studies suggesting that the subclinical phase of MS is the ‘at risk’ period with the greatest susceptibility to systemic inflammation [7,33].

Infectious agents are known to rapidly expand the pool of immune cells, including auto-reactive T-cells in EAE and thus can contribute to the disease progression [5,34,35]. In our study, we detected a substantial increase of B- and T-cell counts in peripheral blood 2 days after the inoculation of bacteria into tissue cages implanted in the animals. Moreover, significant elevation of IL-6 and INF-gamma levels and a tendency to higher levels of TNF-alpha in serum was observed after infection with the *S. aureus* wild-type strains at the end of the experiment. However, at this time point the pro-inflammatory cytokine pattern was not detected in animals that were infected with the Eap-deficient strain compared to the control. Intriguingly, in spite of ongoing inflammation in the periphery the infection with *S. aureus* prevented the onset of clinical EAE symptoms and decreased the severity of autoimmune optic neuritis.

The entry of leukocytes into the CNS is considered an early phenomenon in MS that induces blood-brain barrier breakdown and neuroinflammation. The relevance of vascular cell adhesion molecule 1 (VCAM-1) and intercellular adhesion molecule (ICAM-1) for migration of lymphocytes across the blood-brain barrier during EAE has been shown in several experimental studies [36-38]. Although the very late antigen-4 and vascular cell adhesion molecule 1 (VLA-4-VCAM-1) interaction predominantly mediates the initial adhesion to the vessels, lymphocyte function-associated antigen 1 and intracellular adhesion molecule-1(LFA-1-ICAM-1) are involved in the subsequent trans-endothelial migration of T-cells [39,40].

*S. aureus* expresses anti-adhesive and antimigratory factors that specifically interfere with every step of host inflammatory cell recruitment [41]. A major anti-adhesive protein of the *S. aureus* ‘secretable expanded repertoire adhesive molecules’ (SERAM) is Eap, which is present in 97% of clinical isolates [42]. Eap interacts specifically with endothelial ICAM-1, leading to inhibition of the ICAM-1-integrin interaction [22]. The anti-inflammatory property of Eap in autoimmune diseases has been found in EAE and an animal model of psoriasis [24,40]. These observations led us to hypothesize that the anti-inflammatory effects of *S. aureus* infection in MOG-induced EAE are mediated through Eap secretion. In our study, infection with an Eap-deficient *S. aureus* strain led to increased inflammation, demyelination, and axonal damage in the optic nerve when compared to animals infected with wild-type *S. aureus*. However, histopathological analysis of the optic nerve revealed that the anti-inflammatory property of *S. aureus* infection was not completely abolished by the lack of Eap. Moreover, the infection with the Eap-deficient strain prevented the clinical manifestation of the disease indicating that additional factors, such as enterotoxins secreted by bacteria might be involved in the anti-inflammatory effects of *S. aureus*. However, the dual role of these enterotoxins has been observed in previous EAE-studies [30,43-45].

Given the importance of ICAM-1 antagonism in different autoimmune diseases [46], Eap is believed to be the most potent factor for inhibition of T-cell migration into the CNS. This hypothesis is supported by our histopathological evaluations, which revealed a substantial reduction in the number of lymphocytes in inflammatory infiltrates obtained from the optic nerves of wild-type *S. aureus*-infected animals. In our previous EAE studies, we observed that a strong anti-inflammatory response is not always sufficient to prevent neurodegeneration [12,15,47]. Although the increased neuronal and axonal survivals detected in *S. aureus*-infected animals seemed to be related to the anti-inflammatory properties of Eap, we cannot rule out the possibility of direct neuroprotection by Eap. Further studies are needed to clarify this issue.

Approval of immunosuppressive drugs for MS treatment has progressed very rapidly in recent years. Thus, the incidence of infections in MS patients and the problems associated with them are increasing. Our results demonstrate, for the first time, that chronic *S. aureus* infection has beneficial effects on EAE. Importantly, our results do not trivialize the necessity for antibiotic treatment of infections. As infections activate peripheral inflammatory events, they can induce a general immune response in MS patients known as ‘sickness behavior’. However, some factors released by bacteria seem to have a beneficial effect on autoimmune inflammation in the CNS.

## Conclusions

Bacterial infections have been assumed to worsen MS disease symptoms and to lead to increased neurodegeneration. In contrast, our results demonstrate for the first time that chronic infection with *S. aureus* induces strong peripheral systemic inflammation, but still prevented clinical symptoms in an animal model of MS. Moreover, *S. aureus* infection reduced autoimmune inflammation of the CNS and reduced the severity of autoimmune optic neuritis. Furthermore, using an Eap-deficient *S. aureus* strain, we showed that secretion of Eap by *S. aureus* plays a major role in preventing autoimmune inflammation of the CNS. Infection with *S. aureus* also increased the number of surviving retinal ganglion cells and their axonal counts, most likely via the anti-inflammatory properties of Eap. However, further studies are needed to determine whether Eap exerts a direct neuroprotective effect.

## Abbreviations

ATCC: American type culture collection
BSA: bovine serum albumin
CD: cluster of differentiation
CFU: colony-forming unit
CNS: central nervous system
EAE: experimental autoimmune encephalomyelitis
Eap: extracellular adherence protein
EDTA: ethylenediaminetetraacetic acid
FG: fluorogold
FACS: fluorescence-activated cell sorting
ICAM-1: intracellular adhesion molecule-1
LFA-1: lymphocyte function-associated antigen 1
MOG: myelin oligodendrocyte glycoprotein
MS: multiple sclerosis
ON: optic nerve
PBS: phosphate buffered saline
Pi: postimmunization
RBC: red blood cells
RGCs: retinal ganglion cells
*S. aureus*: *Staphylococcus aureus*
TCF: tissue cage fluid
